# Medical awareness concerning primary immunodeficiency diseases in the city of São Paulo, Brazil

**DOI:** 10.1590/S1679-45082013000400013

**Published:** 2013

**Authors:** Ellen de Oliveira Dantas, Carolina Sanchez Aranda, Fernanda Aimée Nobre, Kristine Fahl, Juliana Themudo Lessa Mazzucchelli, Erika Felix, Dora Lisa Friedlander-Del Nero, Victor Nudelman, Flavio Sano, Antonio Condino-Neto, Elaine Damasceno, Beatriz Tavares Costa-Carvalho

**Affiliations:** 1 Universidade Federal de São Paulo, São Paulo, SP, Brazil; 2 Hospital Israelita Albert Einstein, São Paulo, SP, Brazil; 3 Hospital Servidor Público Municipal, São Paulo, SP, Brazil; 4 Hospital Nipo-Brasileiro, São Paulo, SP, Brazil; 5 Universidade de São Paulo, São Paulo, SP, Brazil

**Keywords:** Immunodeficiency syndromes, Questionnaires, Knowledge, attitudes and practice in healthcare

## Abstract

**Objective:**

To evaluate medical knowledge of primary immunodeficiency in the city of Sao Paulo (SP).

**Methods:**

A 14-item questionnaire about primary immunodeficiency was applied to physicians who worked at general hospitals. One of the questions presented 25 clinical situations that could be associated or not with primary immunodeficiency, and the percentage of appropriate answers generated a knowledge indicator.

**Results:**

Seven hundred and forty-six participated in the study, among them 215 pediatricians (28.8%), 244 surgeons (32.7%), and 287 clinicians (38.5%). About 70% of the physicians responded that they had learned about primary immunodeficiency in graduate school or in residency training. Treatment of patients that use antibiotics frequently was reported by 75% dos physicians, but only 34.1% had already investigated a patient and 77.8% said they did not know the ten warning signs for primary immunodeficiency. The knowledge indicator obtained showed a mean of 45.72% (±17.87). Only 26.6% if the pediatricians and 6.6% of clinicians and surgeons showed a knowledge indicator of at least 67% (equivalent to an appropriate answer in two thirds of the clinical situations).

**Conclusion:**

There is a deficit in medical knowledge of primary immunodeficiency in the city of Sao Paulo, even among pediatricians, despite having greater contact with the theme over the last few years. The improvement of information on primary immunodeficiency in the medical community is an important step towards the diagnosis and treatment process of these diseases.

## RESUMO

**Objetivo:**

Avaliar o conhecimento médico sobre as imunodeficiências primárias na cidade de São Paulo (SP).

**Métodos:**

Um questionário de 14 questões sobre as imunodeficiências primárias foi aplicado a médicos que trabalhavam em hospitais gerais. Uma das questões apresentava 25 situações clínicas que poderiam ou não estar associadas às imunodeficiências primárias, e a porcentagem de respostas apropriadas gerou um indicador de conhecimento.

**Resultados:**

Participaram do estudo 746 médicos, dentre os quais 215 pediatras (28,8%), 244 cirurgiões (32,7%) e 287 clínicos (38,5%). Cerca de 70% dos médicos responderam ter aprendido sobre as imunodeficiências primárias na graduação ou na residência médica. O atendimento a pacientes que usam antibióticos com frequência foi relatado por 75% dos médicos, mas apenas 34,1% já haviam investigado algum paciente e 77,8% não conheciam os dez sinais de alerta para as imunodeficiências primárias. O indicador de conhecimento obtido apresentou uma média de 45,72% (±17,87). Apenas 26,6% dos pediatras e 6,6% tanto dos clínicos quanto dos cirurgiões apresentaram indicador de conhecimento de pelo menos 67% (equivalente à resposta apropriada em dois terços das situações clínicas).

**Conclusão:**

Há uma deficiência no conhecimento médico das imunodeficiências primárias na cidade de São Paulo, mesmo entre os pediatras, a despeito do maior contato com o tema nos últimos anos. A melhora da informação sobre as imunodeficiências primárias entre a comunidade médica é um importante passo para o diagnóstico e o tratamento precoces dessas doenças.

## INTRODUCTION

Primary immunodeficiency (PIM) represents more than 200 genetic diseases, in which alterations in the immune system (IS) cause greater predisposition towards infections, autoimmune phenomena, allergies, and neoplasms^(1)^. Although individually considered rare, the estimated frequency varies from 1:2,000 to 1:10,000 as a group^(2,3)^. It is known, however, that most of these diseases have not yet been described^(4,5)^.

Over the last decades, studies have allowed a greater understanding of the pathophysiology of PIM, which enables greater diagnostic precision, as well as the indication of new treatment strategies^(6,7)^. On the other hand, the large diversity of genetic defects and clinical manifestations makes recognition and diagnosis of patients with PIM a challenge^(8)^.

Serious immune defects are more frequently recognized, but there are cases in which PIM is diagnosed only when the patient is submitted to one or more hospitalizations and may already present with permanent sequelae^(9,10)^.

The lack of medical knowledge is identified in many countries as the probable cause for late diagnosis and underdiagnosis of PIM^(11,12)^.

## OBJECTIVE

To evaluate the medical knowledge regarding primary immunodeficiency diseases in the city of São Paulo.

## METHODS

This is a cross-sectional study conducted in seven general hospitals (three public and four private) in the city of São Paulo (SP), during the period from July 2008 to January 2010.

The choice of hospitals took into consideration services rendered in the large medical specialties (clinical, surgical, and pediatrics), as well as the possibility of access by the researchers, considering the large dimensions of the city.

Considering that in 2009 there were 62,896 registered physicians in São Paulo ^(13)^, for a simple random sample, with a 95% confidence interval (95%CI), 363 questionnaires would be necessary, with a sampling error of 5% at most. We opted for doubling this number, considering that the sample selection would be by convenience, reaching the number of 726 questionnaires, thus involving approximately 1% of the physicians with active Medical Registry Council (CRM) numbers in the city.

The inclusion criteria for the physician in the study were signing of the informed consent form and having been an assistance worker in previously selected hospitals as a day-shift employee or a physician on call.

Exclusion criteria were refusal to participate in the study and possible prior participation in another hospital. The study was approved by the Research Ethics Committee of the *Universidade Federal de São Paulo* (UNIFESP), under approval number CEP 0793/09.

The instrument used to collect data was a questionnaire drawn up by immunologists.

The first part of the questionnaire refers to participant identification, by means of name (optional), time since graduation, specialization, and place of work (public or private; in an ambulatory, ward/infirmary and/or emergency). The second part had 14 closed questions: “Did you learn anything about PIM at the university or during medical residency?”; “After finishing your residency training, did you hear about or learn anything about PIM?”; “Have you seen patients with recurring infections?”; “Do you treat patients who frequently use antibiotics?”; “Did you know that patients who frequently use antibiotics may have PIM?”; “Have you ever investigated any of your patients regarding PIM?”; “If you want to evaluate a patient with a suspect of PIM and need instructions as to the laboratory tests, do you know who to ask?”; “Do you think that every patient with PIM is seriously ill?”; “Did you know that there is treatment for PIM?”; “Did you know that patients with PIM should not receive vaccinations with live microorganisms?”; “What is your greatest difficulty in evaluating your patient's IS?”; “Which of the following clinical situations make you think of PIM?” (This question contained 25 items); “Do you know the ten PIM warning signs?”; “After reading the signs of warning, do you think that any of your patients needs to be investigated for PIM?”

Each participant received a card that described the ten PIM warning signs drawn up by the Jeffrey Modell Foundation (JMF) and adapted by the Brazilian Group for Immunodeficiency (BRAGID) to be read before responding to the last question^(14,15)^.

The databank was prepared on Excel software and statistical analyses were done with the help of the Statistical Package for Social Sciences (SPSS Inc., Chicago, USA) program, version 17.0. For all the statistical tests, a 5% level of significance was used.

Initially, the data were analyzed descriptively. For the categorical variables, absolute and relative frequencies were presented. For numerical variables, summary measurements were presented (mean, minimum, maximum, and standard deviation). To verify an association among categorical variables, the χ^2^ test was used.

A knowledge indicator (KI) was constructed in the form of percentage, by the sum of scores of the 25 clinical situations presented in one of the questions. One point was attributed for each answer considered adequate. The comparison of means between the two groups was performed via Student's *t* test for independent samples. To compare means among more than two groups, variance analysis (ANOVA) was used. ANOVA presupposed the normality of the data, which was verified by the Kolmogorov-Smirnov test. When there were signs of lack of normality, Kruskal-Wallis's non-parametric test was used.

Finally, a multiple linear regression model was adjusted to simultaneously verify which of the variables chosen influenced the KI on PIM. The model had KI as a dependable variable. Explanatory variables were considered: specialty, year of graduation, link to a teaching institute, scope of specialty (public or private service, or both) and the answer given (yes or no) to some selected questions (“Do you see patients who frequently use antibiotics?”; “Did you know that patients who frequently use antibiotics may have PIM?”; “Have you ever investigated one of your patients regarding PIM?”; “Do you know the ten warning signs of PIM?”). The linear regression model has the presupposition of data normality, which was verified by means of the Kolmogorov-Smirnov test for normality in the residual (difference between the value used and estimated by the model) after adjustment of the model. For this, the standardized and studentized residual was used. Additionally, verification of the existence of points of influence was made via Cook's D criterion. At first, a complete model was adjusted, with the inclusion of all the independent variables. Next, non-significant variables were eliminated one by one (backward method).

## RESULTS

All the invited hospitals accepted participation in the study. Seven hundred and sixty-one physicians were approached, of which 746 (98%) responded to the questionnaire distributed among pediatricians, clinicians, and surgeons.

More than 50% of the participants had some form of link with teaching institutions, had concluded graduate school after the year 2000, and worked both in public and private services (Table 1).

**Table 1:** Distribution of the physicians by specialty, link with a teaching institution, year of graduation, and sphere of activity

Characteristics		n (%)
Specialty		746 (100.0)
	Clinical area	287 (38.5)
	Surgery	244 (32.7)
	Pediatrics	215 (28.8)
Link with teaching institution		746 (100.0)
	No	277 (37.1)
	Yes	469 (62.9)
Year of graduation		704 (100.0)
	Up to 1979	49 (7.0)
	1980-1989	112 (15.9)
	1990-1999	174 (24.7)
	After 2000	369 (52.4)
	No answer	42
Sphere of activity		680 (100.0)
	Public service	204 (30.0)
	Private service	102 (15.0)
	Both	374 (55.0)
	No answer	66

### Knowledge about primary immunodeficiency

Knowledge about PIM during graduation and medical residency increased progressively with the year of graduation, reaching 78.4% among those who graduated after the year 2000 (p<0.0001).

As to medical specialties, the pediatricians affirmed having had greater contact with information regarding PIM up until the end of their medical residency training (82.2%), compared to the clinicians (70.3%) and surgeons (50.3%), with p<0.0001. After specialization, the pediatricians responded that they had heard of PIM in a greater percentage (70.7%) than the clinicians (43.8%) and surgeons (33.8%), with p<0.0001.

### Care of patients with recurring infection

The care of patients with recurring infections was made by 69.4% of the professionals, with a greater percentage among the pediatricians (82.1%) than among the clinicians (67.4%) and surgeons (60.7%), with p<0.0001.

Of the total number of physicians, 78.5% responded that they cared for patients who used antibiotics frequently (question 4), and 74.4% said they knew that these patients could have PIM (question 5), with a greater percentage among the pediatricians (88.8%) than among the clinicians (74.9%) and surgeons (61%), and p<0.0001. However, when asked if they had ever investigated a patient as to PIM, only 34.1% responded affirmatively, a percentage that increased to 49% among those who answered “yes” to questions 4 and 5 (p<0.0001).

### Difficulty in investigating immunosuppression

Approximately 55% of the clinicians and surgeons identified unfamiliarity of the specialist as the greatest difficulty in investigating the patient's IS, while 63.3% of the pediatricians pointed out the laboratory. Cost was chosen as the greatest difficulty by 36.7% of the physicians, and 0.3% responded “others”.

### Knowledge about primary immunodeficiency

The percentage of answers given to questions related to PIM may be observed on table 2, and its distribution was not homogenous among the specialties (p<0.05).

**Table 2:** Distribution of physicians by questions related to primary immunodeficiency according to specialty

Questions		Specialty			Total (%)
		Clinical area (%)	Surgery (%)	Pediatrics (%)	
If you want to evaluate a patient with suspected PIM and desire guidance about laboratory tests, do you know who to ask?*					
	No	36.8	58.7	25.1	40.6
	Yes	63.2	41.3	74.9	59.4
Do you think every patient with PIM is seriously ill?**					
	No	80.5	76.1	89.2	81.6
	Yes	19.5	23.9	10.8	18.4
Did you know that there is treatment for patients with PIM?*					
	No	33.2	39.1	17.8	30.7
	Yes	66.8	60.9	82.2	69.3
Did you know that patients with PIM should not receive vaccinations with live microorganisms?*					
	No	30.4	45.9	17.3	31.7
	Yes	69.6	54.1	82.7	68.3

* p<0.0001;

** p=0.0013; PIM: primary immunodeficiency.

In one of the questions, 25 clinical situations were given, 19 of which could lead the physician to think of PIM. The percentage of answers appropriate for those related to PIM may be seen on table 3.

**Table 3:** Knowledge about situations that could be related to primary immunodeficiency

Questions	Knowledge (%)
Ventricular septum defect	8.5
Neonatal tetanus	11.6
Late umbilical cord detachment (>3 weeks)	15.1
Absent tonsil	27.1
Polyendocrinopathy	30.4
Poliomyelitis after Sabin vaccination	40.5
BCG-Osis	41.6
Delayed wound healing	42.1
Bronchiectasis	43.1
Lymphoma	43.3
Chronic diarrhea due to giardiasis	46.5
Serious periodontitis	47.3
Difficulty in gaining weight	49.9
2 pneumonias in 1 year	55.6
Autoimmune cytopenias	56.1
5 otitis events in 1 years	71.7
Sepsis by atypical bacteria	76.8
Persistent candidiasis	80.2
Infections by opportunistic microorganisms	84.2

The KI calculated in this project showed a mean of 45.72% (standard deviation: 17.87%), equivalent to the knowledge of 11 to 12 items, and a median of 48%. The minimum value observed was 0%, and the maximum was 88%.

The mean KI of the pediatricians (55.26%) was greater than that of the clinicians (43.96%), followed by the surgeons (39.48%), with p<0.0001. No differences were seen in the KI among physicians linked or not to teaching institutions nor according to year of graduation.

In multiple linear regressions, the effect of independent variables chosen over the KI can be seen on table 4.

**Table 4:** Coefficients of the multiple linear regression model of the Knowledge Indicator (final model)

Variables		Linear regression coefficient (95% CI)	p value
Did you learn about PIM in university or during medical residency?			
	Yes	5.37	<0.0001
Do you see patients that frequently use antibiotics?			
	Yes	4.46	0.0060
Did you know that patients who use antibiotics frequently may have PIM?			
	Yes	8.51	<0.0001
Have you ever investigated one of your patients for PIM?			
	Yes	3.43	0.0220
Do you know the 10 warning signs for suspected PIM?			
	Yes	4.51	0.0050
Type of activity			
	Public	2.65	0.0540
	Private	ns	ns
Specialty			
	Pediatrics	9.22	0.0001
	Surgery	ns	ns
Link with a teaching institution			
	Yes	ns	ns
Year of graduation			
	Up to 1979	-6.18	0.0160
	1980-1989	ns	ns
	1990-1999	ns	ns
	Constant	27.13	<0.0001

95%CI: 95% confidence interval; PIM: primary immunodeficiency; ns: not significant.

The percentage of physicians that had a KI ≥66.7% was analyzed, which corresponded to approximately two-thirds of correct questions. Only 12.3% of the physicians reached this index of correct answers; when specialties were evaluated, the percentage was 26.4% among pediatricians, and 6.6% among clinicians and surgeons.

### Warning signs of primary immunodeficiency

Only 22.2% of the total of physicians knew the ten warning signs for PIM, with no significant difference between time since graduation or link to teaching institutions (p>0.05), but among the pediatricians, knowledge was greater (43.9%) than among the clinicians (13.03%) and surgeons (14.52%), with p<0.0001.

After reading the warning signs, 72.6% of the pediatricians and 57.6% of both clinicians and surgeons answered that at least one of their patients would need to be investigated for PIM.

## DISCUSSION

Early diagnosis offers the best opportunity for the introduction of adequate treatment and reduced morbimortality of PIM^(11)^. Specialists estimate that between 70 and 90% of PIM remain without a diagnosis, which leads us to believe that the presence of affected patients is more common that what was previously thought^(5)^.

Despite lack of knowledge being pointed out as one of the factors responsible for delayed diagnosis and underdiagnosis of PIM, there are few studies that evaluate this datum objectively, which were carried out in other countries – and most of them among pediatricians^(16-19)^.

The number of recognized PIM cases has grown significantly over the last few years^(8)^, which may have contributed towards greater learning during graduate school or medical residency among those who graduated after the year 2000. Additionally, most centers specialized in the diagnosis and treatment of these diseases are located in São Paulo^(20)^.

The sparse knowledge about the warning signs for PIM, such as was noted among the physicians in this investigation, as well as the lack of information as to the treatments available, are factors with direct consequences on the lack of recognition and diagnosis of these diseases^(2)^. At the immunology service of UNIFESP, we observe that patients with common variable immunodeficiency (CVID) showed a mean time of delayed diagnosis of 6.7 years^(21)^. Even in developed countries, the diagnosis is often made after many years of symptoms, when the patient is hospitalized or when he/she already presents with sequelae from the infections^(22)^. Detailed protocols have been published with clinical and laboratorial information to facilitate the diagnosis and initiation of investigation by the non-immunologist physician^(23,24)^. However, it is not known how much of this information is really put into practice.

In this study, most of the physicians affirmed seeing patients who frequently use antibiotics and knowing that these patients might have PIM. It was expected that the percentage of participants that had indicated some form of investigation for PIM would also be high, which did not happen. Maybe the idea that PIM is extremely rare, along with difficulty in the investigation, continue to contribute towards PIM still not being placed by the physician among the initial differential diagnoses of a patient, even if at some time during medical training he/she learned something about the subject, reinforcing the concept that medical education should be on-going so that there is greater recognition of these diseases^(25)^.

Vaccines constituted by live or attenuated microorganisms are generally contraindicated in patients with PIM, especially in cases of serious deficiencies, due to a greater risk of adverse effects^(11)^. It is clear that pediatricians are better informed about this aspect than clinicians and surgeons, and that they are the professionals that generally prescribe vaccination for the child. Despite this, almost all the patients with PIM seen at UNIFESP received all the vaccinations recommended for their age by the Ministry of Health^(21)^. A clinician or obstetrician, however, should be apt to instruct a pregnant woman with a family history suggestive of immunodeficiency (ID) that for the child, once born, vaccination with the Calmette-Guérin bacillus (BCG) should be delayed until its immunocompetence is defined^(26)^.

The adverse effect of the BCG vaccine was related to PIM by only 41.6% of the physicians, despite being an important warning sign in Brazil, where this vaccine is recommended to newborns weighing ≥2.000g as soon as possible, preferentially while still in the nursery^(27)^. In patients with severe combined immunodeficiency (SCID) or leukocyte mycobatericidal defect, dissemination of the vaccine bacillus occurs with a relevant frequency^(28)^.

The clinical characteristics that were least related to PIM were ventricular septal defect (8.5%) and neonatal tetanus (11.6%), associated with the DiGeorge Syndrome (DGS)^(29)^. This syndrome has an approximate incidence of 1:3,000 liveborns, but it is still rarely diagnosed in Brazil, which contributes with only four recorded patients among a total of 116 DGS cases presented in the last publication of the Latin American Association for Immunodeficiencies (LASID)^(12)^.

In Turkey, 786 questionnaires with 71 items were distributed to pediatricians. Data from the anamnesis and physical examination, with a family history positive for PIM, early death in the family, chronic diarrhea due to giardiasis, recurring oral aphtous stomatitis, absence of tonsils, and delay in weight and height development, were related to PIM by more than 60% of those interviewed. On the other hand, neonatal tetanus, liver abscess, and poliomyelitis after vaccination with an attenuated virus were not related to PIM^(16)^. In our study, chronic diarrhea due to giardiasis, absence of tonsils, delayed weight and height development, and poliomyelitis after the Sabin vaccine were considered suggestive of PIM by less than 50% of the physicians, calling attention to the little contact of the participants with clinical aspects of PIM when they differentiate them from recurring infections.

As to the calculated KI, the mean was greater among the pediatricians, followed by the clinicians and surgeons. Considering that a desirable KI would be 66.6% (equivalent to the appropriate answer to two-thirds of the clinical situations), the results of this study were lower than expected. However, this result was very similar to that observed in Kuwait, where 26% of the pediatricians responded correctly to two-thirds of the items on a questionnaire to evaluate knowledge about PIM^(17)^.

The year of graduation before 1979 was associated with a decreased KI. The advent of the acquired immunodeficiency syndrome (AIDS) triggered a greater search for understanding of IS, which turned the attention of physicians towards diseases that evolve with alterations of defense mechanisms and the consequent association with other diseases, including PIMs. In Brazil, the increased number of cases of AIDS diagnosed as of the 1980s may explain less familiarity with the IDs among those that graduated before this decade^(30)^.

Specialization in Pediatrics was one of the factors associated with an increase in KI, probably due to the fact that most PIMs can be diagnosed in childhood. Maybe there is less emphasis on the study of PIM on the part of clinicians and surgeons due to the idea that these diseases do not affect the adult individual. However, the first symptoms and the diagnosis may occur only during the adult phase. Additionally, therapeutic advances afford patients a greater possibility of survival^(24)^. In CVID, the symptoms characteristically begin between the second and fourth decades of life, and it is one of the most common PIMs^(11)^. Other rarer diseases, typical of childhood, may also be detected in the adult as a partial deficiency of adenosine deaminase and DGS^(10)^. Medical specialties that treat adult patients should, therefore, also be targets of programs of divulging information on PIM.

Other factors associated with an increase in KI are related primarily to education and updating information. Considering the need for medical updating in reference to PIMs, the JMF established a program of medical education and public knowledge in 2003: PEPAC – the Physician Education and Public Awareness Campaign. The result of the program was evaluated in 39 countries and there was a significant increase in referrals, diagnoses, and treatments of PIM. In São Paulo, JMF opened the first center for PIM diagnosis and management in 2009. One of the objectives is to support dissemination of knowledge about these diseases among physicians, other professional in the healthcare area, and the lay population^(30)^.

Brazil is part of LASID, which has been registered for PIM as of April 2009, with approximately 700 registered Brazilian patients up until June of 2012 (http://imunodeficiencia.unicamp.br:8080). This number is lower than expected, if one takes into consideration the size of the population and the estimated prevalence of PIM^(12)^.

## CONCLUSION

We conclude that there have been deficiencies in knowledge on the part of physicians as to PIM in the city of São Paulo, even among pediatricians, despite greater contact with the theme over the last few years.

In the absence of triage tests for any phase of life regarding these diseases in our country, one of the main steps to attaining early diagnosis is greater recognition by physicians of the warning signs for investigation of primary immunodeficiency.
